# ^99**m**^ **T** **c**-DTPA Study to Validate an Experimental Model of Ureteral Obstruction in Rabbits: Preliminary Results

**DOI:** 10.1155/2013/929620

**Published:** 2013-12-29

**Authors:** Marcelo Lopes de Lima, Rodolfo Bertti, Juliano César Moro, Fábio Coltro Neto, Ricardo Miyaoka, Adriano Fregonesi, Mariana da Cunha Lopes de Lima, Celso Darío Ramos

**Affiliations:** ^1^Division of Urology, Department of Surgery, Campinas State University, Avenida Vital Brasil 251, Caixa Postal 6142, Cidade Universitária Zeferino Vaz, 13083-888 Campinas, SP, Brazil; ^2^Nuclear Medicine Division, Department of Radiology, Campinas State University, Avenida Vital Brasil 251, Caixa Postal 6142, Cidade Universitária Zeferino Vaz, 13083-888 Campinas, SP, Brazil

## Abstract

*Objective*. To create a ureteral obstruction experimental model that can be proved through ^99m^Tc-DTPA renal scintigraphy and histopathological studies, without causing total renal function loss. *Materials and Methods*. Ten New Zealand white rabbits were submitted to a surgical experiment to create a model of unilateral obstruction to urinary flow. Surgery procedure provided unilateral ureteral obstruction (left kidney) to urinary flow and posteriorly was evaluated by ^99m^Tc-DTPA renal scintigraphy and histopathological study. ^99m^Tc-DTPA renal study was performed to detect and quantify signs of obstruction and to evaluate renal function. Statistical analysis was performed through the Student *t*-test with a significance level of *P*<0.05. *Results*. Nine of the ten rabbits presented left renal unit obstruction and one nonobstructive on the ^99m^Tc-DTPA and histopathological studies. All the right renal units, which were not submitted to surgical procedure, were nonobstructed by the studies. There was a general agreement between scintigraphy and histopathological results in both groups. *Conclusion*. The experimental model promoted the creation of ureteral obstruction in rabbits, confirmed by nuclear medicine scintigraphy and histopathology, and could be used in further studies to better understand urinary obstruction.

## 1. Introduction

Pelvi-ureteric junction (PUJ) obstruction is one of the most frequent congenital anomalies of the urinary tract system. It is associated with pain, hydronephrosis, urinary tract infections, and eventually loss of renal function [1, 2]. It affects around 40% to 60% of all newborns with hydronephrosis [3], two times more common in males, and may be bilateral in 5% to 15% of cases [1, 4].

PUJ obstruction may be caused by intrinsic factors, like aperistaltic ureteral segment, obstructive fold mucosa, ureteral polyp, or ureteral stenosis [5]. Among extrinsic factors stands inferior renal polar vessel crossing anteriorly the PUJ [6].

Treatment varies from clinical observation to surgery. There are some surgical modalities available to correct the PUJ obstruction, differing from the open pyeloplasty to the latest in technology such as robotic assisted surgeries and endourological procedures [7–9].

Currently scientific literature is short in ureteral obstruction models that accurately reproduce the clinical and microscopic features of this infirmity [10–12].

The creation of a standardized experimental model that would be able to cause obstructive disturbance without leading to renal function loss would provide information capable of enhancing not only the diagnosis but also the treatment choices.

Through this model, one would be able to test drugs, seek biochemical markers, assess radiopharmaceutical produces, research pathological features related to ureteral obstruction, and evaluate therapeutic methods.

The objective of the present study is to create a ureteral obstruction experimental model that can be proved through renogram with technetium-99m diethylenetriaminepentaacetic acid (^99m^Tc-DTPA) and histopathological studies, without causing total renal function loss.

## 2. Materials and Methods

The protocol for the research project was approved by the institution research and ethical commission, within which the work was undertaken.

After approval by the research and ethical commission, ten New Zealand white rabbits were submitted to the present surgical experiment. The animals were all 3 months old and female and weighted 3.5 Kg on average.

After general anesthesia acquired with intramuscular injection of ketamine (30 mg/kg) and xylazine (5 mg/kg), the rabbits' abdominal cavities were opened through a midline abdominal incision of 15 cm in length and their left ureter was identified and dissected out, preserving its vascularization. Subsequently, a 1.5 cm long incision was made into the left psoas muscle starting 1.5 cm below the inferior pole of left kidney, creating a longitudinal groove.

**Figure 1:**
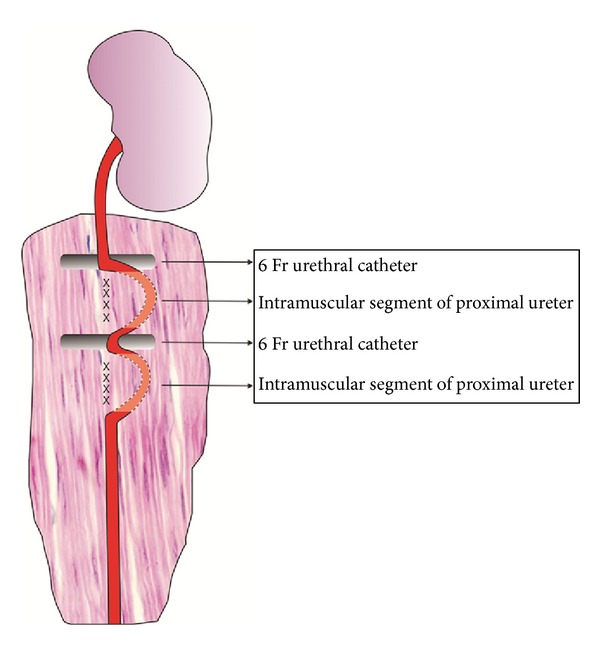
Surgical technique model, “W” shape.

One small segment of a 6 Fr silicon catheter was perpendicularly fixed in the middle of this groove through polypropylene 4.0 thread sutures. After that, the proximal ureter segment was gently inserted into the muscular groove at three distinct points, leaving a “W” shape (Figure 1) with two parts in the groove and one central part free, over the catheter, for ureteral perfusion control. The proximal ureter segment was kept into the muscular groove through suture with polypropylene cord. The 6 Fr silicon catheter was used to reproduce the circumvolutions of the ureter (Figure 1). The right ureter was identified and dissected to be used as the control. Care was taken not to jeopardize the ureteral vascularization. The cavity was then sutured with nylon 2.0.

After recovery from the surgical procedure the animals were sent back to individual cages, where they were thoroughly followed up for 30 days. On the 30th day after surgical procedure, a ^99m^Tc-DTPA renal study with furosemide was performed in all animals, under general anesthesia, to detect and quantify signs of obstruction. Afterwards, all the animals were sent back to the laboratory and sacrificed. The renal units were sent to histopathological evaluation.

### 2.1. Radiopharmaceutical Preparation

The radiopharmaceutical was reconstituted in accordance with the manufacturer's instructions (IPEN DTPA agent, São Paulo, Brazil; IPEN Molybdenum generator (Mo-99-Tc^99m^), São Paulo, Brazil), calculating the dose according to the animal's weight (3.7 MBq per kilogram of body weight) [13].

A routine bladder catheterization was performed. A distended bladder could create enough back pressure on the ureter to give a false impression of obstruction.

Diuretic time-activity curve was obtained based on the dynamic images acquired for 20 minutes after furosemide injection (1 mg/kg, maximum dose of 20 mg) [13, 14].

The qualitative analysis was performed evaluating the renal blood flow and the uptake, concentration, and excretion of the radiopharmaceutical by the kidneys.

In the present study, the determination of the percentage of the radiopharmaceutical excreted was adapted from the method of drainage *T*1/2 [14]. The study was considered obstructive when excretion was below 50% in 20 minutes, corresponding to a *T*1/2 above 20 minutes; nonobstructive when excretion was above 60% in 20 minutes (*T*1/2 < 15 minutes); and indeterminate when excretion was between 50% and 60% at 20 minutes [15].

The excretion estimative of ^99m^Tc-DTPA by the kidneys was statistically analyzed through the Student *t*-test with a significance level of *P* < 0.05 which was considered statistically significant.

## 3. Results

Nine of the ten rabbits presented left renal unit obstruction on the ^99m^Tc-DTPA study and one nonobstructive (Table 1), with mild decreased renal function in two obstructed renal units and moderate decreased function in seven obstructed renal units. All the right renal units were nonobstructed on ^99m^Tc-DTPA study, with normal renal function by qualitative analysis.

### 3.1. Macroscopic Study

Findings from both pathological and scintigraphic assessment were concordant. All obstructed kidneys according to scintigraphy presented hydronephrosis. Of these, two units were classified as mild hydronephrosis whilst the remaining seven units were classified as moderate. No specimen presented severe hydronephrosis. Mild hydronephrosis was considered when proximal ureteral segments showed a dilation of 2 : 1 ratio when compared with proximal ureteral diameter of control kidneys. Similarly, moderate hydronephrosis was determined as a 3 : 1 to 4 : 1 ratio dilation. Control kidneys weighed 10 g in average and cortical width varied from 0.5 cm to 0.6 cm. All left side kidneys classified as hydronephrotic (*n* = 9) weighed 13.5 g in average and cortex varied from 0.3 cm to 0.4 cm with an average weight gain of 34.7% (10–55%) when compared to the right side (*P* < 0.05).

### 3.2. Microscopic Study

Parenchymal renal alterations in hydronephrotic specimens were mostly noted in upper and lower poles where composed papillae are found. Medial portion of the kidney where simple papillae predominate was less affected. Renal cortex is minimally changed under microscopic view in mild hydronephrosis: ectasia, tubular atrophy, and mild nephritis. On the other hand, in moderate hydronephrosis, dilation alterations were more obvious: atrophy and ectasia seen in tubules, collecting ducts, and contorted tubules; Tamm-Horsfall protein accumulation in peritubular, tubular, and Bowman's spaces; and chronic nephritis, as previously described in renal obstruction by Heptinstall [16].

Besides the above mentioned alterations, moderate hydronephrotic cases also presented a “thyroidization” process, which is a tubular atrophy development with accumulation of eosinophilic amorphous substance in its lumen, giving it a thyroid gland-like aspect. Within a 30-day period, no signs of severe hydronephrosis could be seen.

Scar tissue (fibroblasts) and inflammatory cells were not observed in ureteral segments specimens that were wrapped by psoas muscle plicature.

## 4. Discussion

PUJ obstruction represents approximately 40% of the urologic diseases diagnosed in the prenatal period. Frequently it does not require invasive treatment, but only clinical and laboratorial followup. In about 20% of the cases, a postnatal surgical correction of the PUJ obstruction is necessary in order to preserve renal function. If left untreated, it may lead to a syndrome with blockade and reduction of the urine flow, dilatation of the urinary tract, hydronephrosis, and other symptoms [1–3]. Several aspects of urinary obstruction have been studied. Urinary tract obstruction results in renal compensatory mechanisms and may cause irrecoverable functional loss and histological alterations. The pathophysiology of this progression is poorly understood [17].

Several factors may interfere in the process of obstruction. Wang et al. [18] have demonstrated that neonatally induced partial unilateral ureteral obstruction in rats is associated with changes in the abundance of renal acid-base transporters that are paralleled by reduction in renal function, depending on the severity and duration of obstruction. MacLellan et al. [17] have demonstrated that urinary tract obstruction is associated with elevated urinary levels of alanine, succinate, dimethylglycine, creatinine, taurine, choline-like compounds, hippurate, and lactate and decreased urinary levels of 2-oxoglutarate and citrate. Despite these reports on biochemical alterations, histological changes during chronic partial ureteral obstruction were not well studied [19]. Long-term results confirmed that partial ureteral obstruction in newborn mice produces fibrotic lesions of the renal parenchyma, however with no correlation with dilatation of the upper tract [20] and substances like atorvastatin ameliorated fibrosis, and helped preserve kidney filtration function [19]. Experimental models in animals would permit testing of new drugs and observation of histological changes in the urinary obstruction.

There are some experimental models of ureteral obstruction [10–12, 21, 22]. However, none have confirmed partial obstruction and the small size of these animals may hinder new surgical techniques evaluations. The objective of the present study was to develop a new experimental model of ureter stenosis, expanding the existing settings, although a bigger animal model was used, as rabbits were included in the research.

The rabbit is an attractive model for kidney studies, including renal obstruction and transplantation, in view of its docility, convenient size, easy maintenance, low cost, and renal anatomical and functional similarity to human kidneys [23–26]. Although the rabbit kidney is unipapillary and the human kidney is multipapillary [27], there have been several studies in the literature that have used rabbits as animal model, evaluating different renal diseases, stating that the rabbit is suitable biological model for lesions of the kidney [26, 27].

It is estimated that rabbit life expectancy is around 8 years. If we were to make a linear interpolation from that figure to human life expectancy, we might say that one rabbit year is roughly equivalent to one human decade [28, 29]. Then, we considered that 30 days were sufficient time to observe changes of obstruction.

The renal scintigraphy was performed to confirm the urinary obstruction and evaluate the renal function maintenance, allowing the validation of the method and permitting the safe use of the model in the future. Considering this similarity and previous studies in the literature [23, 26], we have considered the method of *T*1/2 adequate for rabbits, calculating the adequate doses of DTPA-^99m^Tc and furosemide according to the weight of the animals.

The renal scintigraphy performed in the sample clearly showed that nine renal units presented obstruction. None of the left units showed total loss of function. The only unit without obstruction could be a result of a fail in the creation of circumvolutions. The diagnosis of ureteral obstruction is commonly made by ^99m^Tc-DTPA diuretic renogram [14, 15]. ^99m^Tc-DTPA is one of the most widely used radiopharmaceuticals to assess renal function of patients with suspected urinary obstruction, with excretion predominantly by glomerular filtration. Renal scintigraphy was preferentially used because it is a noninvasive and quick exam, providing rapid dynamic imaging of the kidneys, including renal flow, function, and urinary outflow evaluation [14, 15].

The utilized surgical technique in the present study is a modification of Ulm and Miller's [10] procedure, authors who first described the fixation of the upper ureter to the psoas muscle. Our PUJ model uses circumvolutions of the ureter to promote a local alteration of the peristaltic wave, not an extrinsic constriction, and through these ups and downs, a functional alteration may have occurred in the ureteral emptying. As Hammad et al. [30] have demonstrated, after complete or partial ureteral obstruction, there are immediate, significant changes in the propagation of electrical impulses in the proximal and distal ureter, which are generally less marked after partial obstruction than after complete obstruction.

The present model has the ability to control the degree of obstruction by the tension force applied in the ureter. One can anticipate that when the obstruction is undone, simply by liberating the ureter from the muscle without resections and anastomosis, it will make it easier to analyze what happens to this renal unit while recovering. This finding is of relevance to improve data and knowledge on the behavior of the PUJ obstruction and also on the sensitivity of diagnostic tests with regard to different degrees of obstruction that are difficult to counsel and to treat the patients efficiently. Based on the need for a trusting model of PUJ obstruction the present study aimed to create an ease-to-do upper ureteral obstruction model. To date there have been some studies on PUJ obstruction regarding the creation of an animal model either for better understanding of the disease biology or for surgical training purposes. Studies varied vastly from the technique used to generate the obstruction to the size of animals submitted to the experiments [31]. Moreover, the histological data was not provided by all of those experiments, and the ones that did mention them lacked sustainable findings. This proposed model of partial ureteral obstruction enables chronic evaluation studies of morphological, functional, and histological changes of the obstructed kidney [32]. We believe that further studies could be developed based on the model described and could reference the findings, though preliminary, of the model described here.

## 5. Conclusions

The experimental model developed promoted the creation of ureteral obstruction in rabbits, confirmed by nuclear medicine and histopathological exams, and could be used in further studies to better understand urinary obstruction.

## Figures and Tables

**Table 1:** Results of the scintigraphic study with ^99m^Tc-DTPA.

Rabbits	^ 99m^Tc-DTPA excretion (%)
1	24.0% (O)
2	74.0% (N)
3	40.0% (O)
4	24.0% (O)
5	38.0% (O)
6	30.0% (O)
7	21.0% (O)
8	30.0% (O)
9	35.0% (O)
10	25.2% (O)

^†^N: nonobstructed; O: obstructed.

## Abbreviations

PUJ: Pelvi-ureteric junction
^99m^Tc-DTPA: Technetium-99m diethylenetriaminepentaacetic acid.
